# Radiation effect on viscous flow of a nanofluid and heat transfer over a nonlinearly stretching sheet

**DOI:** 10.1186/1556-276X-7-229

**Published:** 2012-04-22

**Authors:** Fekry M Hady, Fouad S Ibrahim, Sahar M Abdel-Gaied, Mohamed R Eid

**Affiliations:** 1 Department of Mathematics, Faculty of Science, Assiut University, Assiut 71516, Egypt; 2 Department of Science and Mathematics, Faculty of Education, Assiut University, The New Valley 72111, Egypt; 3 Department of Mathematics, University College in Jamoom, Umm Al-Qura University, Makkah 2064, Saudi Arabia

**Keywords:** nanofluid, nonlinearly stretching surface, viscous dissipation, thermal radiation.

## Abstract

In this work, we study the flow and heat transfer characteristics of a viscous nanofluid over a nonlinearly stretching sheet in the presence of thermal radiation, included in the energy equation, and variable wall temperature. A similarity transformation was used to transform the governing partial differential equations to a system of nonlinear ordinary differential equations. An efficient numerical shooting technique with a fourth-order Runge-Kutta scheme was used to obtain the solution of the boundary value problem. The variations of dimensionless surface temperature, as well as flow and heat-transfer characteristics with the governing dimensionless parameters of the problem, which include the nanoparticle volume fraction *ϕ*, the nonlinearly stretching sheet parameter *n*, the thermal radiation parameter *N_R_*, and the viscous dissipation parameter *Ec*, were graphed and tabulated. Excellent validation of the present numerical results has been achieved with the earlier nonlinearly stretching sheet problem of Cortell for local Nusselt number without taking the effect of nanoparticles.

## Background

The problem of viscous flow and heat transfer over a stretching sheet has important industrial applications, for example, in metallurgical processes, such as drawing of continuous filaments through quiescent fluids, annealing and tinning of copper wires, glass blowing, manufacturing of plastic and rubber sheets, crystal growing, and continuous cooling and fiber spinning, in addition to wide-ranging applications in many engineering processes, such as polymer extrusion, wire drawing, continuous casting, manufacturing of foods and paper, glass fiber production, stretching of plastic films, and many others. During the manufacture of these sheets, the melt issues from a slit and is subsequently stretched to achieve the desired thickness. The final product with the desired characteristics strictly depends upon the stretching rate, the rate of cooling in the process, and the process of stretching. In view of these applications, Sakiadis [1,2] investigated the boundary-layer flow of a viscous fluid past a moving solid surface; various aspects of the problem have been explored by many authors in the past decades.

However, all these studies are restricted to linear stretching of the sheet. It is worth mentioning that the stretching is not necessarily linear. In view of this, Kumaran and Ramanaih [3] studied flow over a quadratic stretching sheet, but only a few recent studies focused on exponentially and nonlinearly stretching sheet are cited here. Magyari and Keller [4], Elbashbeshy [5], Khan and Sanjayanand [6], Sanjayanand and Khan [7], Sajid and Hayat [8], and Partha et al. [9] studied the heat transfer characteristics of viscous and viscoelastic fluid flows over an exponentially stretching sheet. Vajravelu [10], Vajravelu and Cannon [11], Cortell [12-15], Prasad et al. [16], Afzal [17], and Nandeppanavar et al. [18] studied the effects of various parameters governing the flow of a viscous fluid over a nonlinearly stretching sheet.

A nanofluid is a new class of heat transfer fluids that contain a base fluid and nanoparticles. The use of additives is a technique applied to enhance the heat transfer performance of base fluids. The thermal conductivity of ordinary heat transfer fluids is not adequate to meet today's cooling rate requirements. Nanofluids have been shown to increase the thermal conductivity and convective heat transfer performance of the base liquids. Nanofluids are suspensions of submicronic solid particles (nanoparticles) in common fluids. The term was coined by Choi [19]. The characteristic feature of nanofluids is thermal conductivity enhancement, a phenomenon observed by Masuda et al. [20]. This phenomenon suggests the possibility of using nanofluids in advanced nuclear systems [21]. A comprehensive survey of convective transport in nanofluids was made by Buongiorno [22], who says that a satisfactory explanation for the abnormal increase of the thermal conductivity and viscosity is yet to be found. He focused on further heat transfer enhancement observed in convective situations. Very recently, Kuznetsov and Nield [23] have examined the influence of nanoparticles on natural convection boundary-layer flow past a vertical plate using a model in which Brownian motion and thermophoresis are accounted for. The authors have assumed the simplest possible boundary conditions, namely those in which both the temperature and the nanoparticle fraction are constant along the wall. Furthermore, Nield and Kuznetsov [24,25] have studied the Cheng and Minkowycz [26] problem of natural convection past a vertical plate in a porous medium saturated by a nanofluid. The model used for the nanofluid incorporates the effects of Brownian motion and thermophoresis for the porous medium. The Darcy model has been employed.

Hamad and Bashir [27] numerically investigated the problem of forced convection heat transfer to the power law non-Newtonian nanofluid from the stretching surface. Khan and Pop [28] focused on the problem of laminar fluid flow, which results from the stretching of a flat surface in a nanofluid. A similarity solution of the steady boundary layer flow near the stagnation-point flow on a permeable stretching sheet in a porous medium saturated with a nanofluid and in the presence of internal heat generation/absorption was theoretically studied by Hamad and Pop [29]. Hamad and Ferdows [30] investigated the heat and mass transfer analysis for boundary layer stagnation-point flow over a stretching sheet in a porous medium saturated by a nanofluid with internal heat generation/absorption and suction/blowing. The problem of laminar fluid flow, which results from the stretching of a vertical surface with variable stream conditions in a nanofluid, was investigated numerically by Kandasamy et al. [31]. Makinde and Aziz [32] studied numerically the boundary layer flow induced in a nanofluid due to a linearly stretching sheet. Hamad [33] examined the convective flow and heat transfer of an incompressible viscous nanofluid past a semi-infinite vertical stretching sheet in the presence of a magnetic field. All these researchers studied the linear stretching sheet in the nanofluid, but only the numerical investigation by Rana and Bhargava [34] studied the steady laminar boundary fluid flow, which results from the non-linear stretching of a flat surface in a nanofluid, and incorporated the effects of Brownian motion and thermophoresis. Also, more recently, Nadeem and Lee [35] investigated analytically the problem of steady boundary layer flow of nanofluid over an exponential stretching surface including the effects of Brownian motion parameter and thermophoresis parameter.

## Presentation of the hypothesis

To the authors' knowledge, no studies have thus far been communicated with regard to the boundary layer viscous flow and heat transfer of a nanofluid past a nonlinearly stretching sheet in the presence of the radiation effect in a one-phase model. The aim of the present paper is therefore to extend the work of Cortell [13] by taking the steady thermal boundary-layer flow with nonlinearly stretching sheet in a nanofluid. The present study is of immediate interest to all those processes which are highly affected with heat enhancement concept, e.g., cooling of metallic sheets or electronic chips, etc. An efficient numerical shooting technique with a fourth-order Runge-Kutta scheme was used to solve the normalized boundary layer equations, and the effects of nanoparticle volume fraction *ϕ*, nonlinearly stretching sheet parameter *n*, thermal radiation parameter *N_R_*, and viscous dissipation parameter *Ec *are described in details and are further presented in tabular form.

## Testing the hypothesis

### Problem formulation

**Figure 1:**
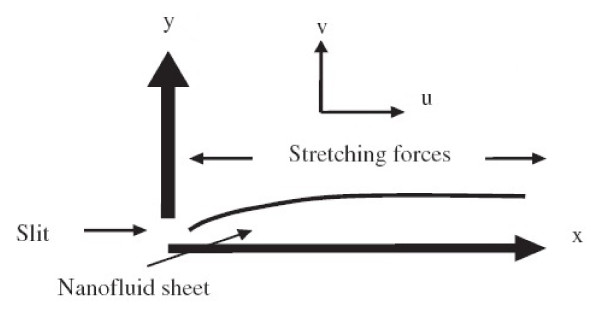
**A schematic diagram of the physical model**.

We consider a steady, incompressible, laminar, two-dimensional boundary layer flow of a viscous nanofluid past a flat sheet coinciding with the plane *y *= 0 and the flow being confined to *y *> 0. The flow is generated due to nonlinear stretching of the sheet caused by the simultaneous application of two equal and opposite forces along the *x*-axis. Keeping the origin fixed, the sheet is then stretched with a velocity *u_w_*(*x*) = *Cx^n^*, where *C *is a constant, *n *is a nonlinear stretching parameter, and *x *is the coordinate measured along the stretching surface, varying nonlinearly with the distance from the slit. A schematic representation of the physical model and coordinate system is depicted in Figure 1. The thermo-physical properties of the nanofluid are given in Table 1 (see [36]). The pressure gradient and external forces are neglected. The basic steady conservation of mass, momentum, and thermal energy equations for nanofluid by using usual boundary-layer approximations in the presence of radiation and viscous dissipation can be written in Cartesian coordinates *x *and *y *as:

**Table 1:** Thermo-physical properties of fluid and nanoparticles (Oztop and Abu-Nada [36]).

Physical properties	Fluid phase (water)	Cu	**Al**_**2**_**O**_**3**_	**TiO**_**2**_
*C_p _*(*J/kgK*)	4179	385	765	686.2
*ρ *(*kg/m*^3^)	997.1	8933	3970	4250
*k*(*W/mK*)	0.613	401	40	8.9538
*β *×10^5 ^(*K*^-1^)	21	1.67	0.85	0.9

(1) $$\frac{\partial u}{\partial x}+\frac{\partial v}{\partial y}=0,$$

(2) $$u\frac{\partial u}{\partial x}+v\frac{\partial u}{\partial y}=\upsilon_{nf}\frac{\partial^{2}u}{\partial y^{2}},$$

(3) $$u\frac{\partial T}{\partial x}+v\frac{\partial T}{\partial y}=\alpha_{nf}\frac{\partial^{2}T}{\partial y^{2}}+\frac{\upsilon_{nf}}{{\left(c_{p}\right)}_{nf}}{\left(\frac{\partial u}{\partial y}\right)}^{2}-\frac{1}{{\left(\rho c_{p}\right)}_{nf}}\frac{\partial q_{r}}{\partial y}.$$

The associated boundary conditions of Equations 1, 2, and 3 can be written as:

(4) $$\begin{array}{c} u=u_{w}\left(x\right)=Cx^{n},v=0;\;\;T=T_{w}\left(x\right)=T_{\infty }+bx^{m}\;\text{at }y=0;\\ u\to 0;\;\;T\to T_{\infty }\;\text{as }y\to \infty , \end{array}$$

where *x *and *y *denote the Cartesian coordinates along the sheet and normal to it, and *u *and *v *are the velocity components of the nanofluid in the *x*- and *y*-directions, respectively. *n *and *m *are the nonlinear stretching parameter and the surface temperature parameter, respectively. The temperature on the wall is *T_w_*, and the ambient is held at constant temperature *T*_∞_. *ρ_nf _*and *μ_nf _*are the density and effective viscosity of the nanofluid, and *α_nf _*and *υ_nf _*are the thermal diffusivity and the kinematic viscosity, respectively, which are defined as (see Khanafer et al. [37]):

(5) $$\begin{array}{c} \upsilon_{nf}=\frac{\mu_{nf}}{\rho_{nf}},\;\;\rho_{nf}=\left(1-\phi \right)\rho_{f}+\phi \rho_{s},\;\mu_{nf}=\frac{\mu_{f}}{{\left(1-\phi \right)}^{2.5}},\;\alpha_{nf}=\frac{k_{nf}}{{\left(\rho c_{p}\right)}_{nf}},\\ {\left(\rho c_{p}\right)}_{nf}=\left(1-\phi \right)\;{\left(\rho c_{p}\right)}_{f}+\phi {\left(\rho c_{p}\right)}_{s},\;\frac{k_{nf}}{k_{f}}=\frac{\left(k_{s}+2k_{f}\right)-2\phi \left(k_{f}-k_{s}\right)}{\left(k_{s}+2k_{f}\right)+2\phi \left(k_{f}-k_{s}\right)}. \end{array}$$

Here, *ϕ *is the solid volume fraction, where *μ_f _*is the viscosity of the basic fluid, *ρ_f _*and *ρ_s _*are the densities of the pure fluid and nanoparticle, respectively, (*ρc_p_*)*_f _*and (*ρc_p_*)*_s _*are the specific heat parameters of the base fluid and nanoparticle, respectively, and *k_f _*and *k_s _*are the thermal conductivities of the base fluid and nanoparticle, respectively. Using the Rosseland approximation for radiation, the radiative heat flux is simplified as:

(6) $$q_{r}=-\frac{4\sigma^{*}}{3k^{*}}\frac{\partial T^{4}}{\partial y},$$

where σ* and k* are the Stefan-Boltzmann constant and the mean absorption coefficient, respectively. We assume that the temperature differences within the flow, such as the term *T*^4^, may be expressed as a linear function of temperature. Hence, expanding *T*^4 ^in a Taylor series about a free stream temperature *T*_∞ _and neglecting higher-order terms, we get:

(7) $$T^{4}≅4T_{\infty }^{3}T-3T_{\infty }^{4}.$$

From Equation 3 and in view of Equations 6 and 7, it is seen that the effect of radiation is to enhance the thermal diffusivity. If we take $N_{R}=k_{nf}k^{*}/\left[4\sigma^{*}T_{\infty }^{3}\right]$as the radiation parameter, Equation 3 becomes:

(8) $$u\frac{\partial T}{\partial x}+v\frac{\partial T}{\partial y}=\frac{\alpha_{nf}}{k_{0}}\frac{\partial^{2}T}{\partial y^{2}}+\frac{\upsilon_{nf}}{{\left(c_{p}\right)}_{nf}}{\left(\frac{\partial u}{\partial y}\right)}^{2},$$

where $k_{0}=\frac{3N_{R}}{3N_{R}+4}$. It is worth citing here that the classical solution for energy equation, Equation 8, without thermal radiation influence can be obtained from the above equation, which reduces to $u\frac{\partial T}{\partial x}+v\frac{\partial T}{\partial y}=\alpha_{nf}\frac{\partial^{2}T}{\partial y^{2}}$as *N_R _*→∞ (i.e., *k*_0 _→ 1) and eliminates viscous dissipation.

By introducing the following non-dimensional variables:

(9) $$\begin{array}{ccc} \eta & =y\sqrt{\frac{C\left(n+1\right)}{2\upsilon_{f}}}x^{\frac{n-1}{2}},\;u=Cx^{n}f^{\prime }\left(\eta \right), & \\ v & =-\sqrt{\frac{C\left(n+1\right)\upsilon_{f}}{2}}x^{\frac{n-1}{2}}\left[f\left(\eta \right)-\frac{n-1}{n+1}\eta f^{\prime }\left(\eta \right)\right],\;\theta \left(\eta \right)=\frac{T-T_{\infty }}{T_{w}-T_{\infty }}, \end{array}$$

then the governing Equations 1, 2, and 8 reduce to:

(10) $$f^{\prime \prime \prime }+{\left(1-\phi \right)}^{2.5}\left(1-\phi +\phi \frac{\rho_{s}}{\rho_{f}}\right)\left(ff^{\prime \prime }-\frac{2n}{n+1}{f^{\prime }}^{2}\right)=0,$$

(11) $$\frac{1}{\text{pr}}\left(\frac{k_{nf}}{k_{f}}\right)\frac{\theta^{\prime \prime }}{k_{0}}+\frac{Ec}{{\left(1-\phi \right)}^{2.5}}x^{2n-m}{f^{\prime \prime }}^{2}+\left[\left(1-\phi \right)+\phi \frac{{\left(\rho c_{p}\right)}_{s}}{{\left(\rho c_{p}\right)}_{f}}\right]\left(f\theta^{\prime }-\frac{2m}{n+1}f^{\prime }\theta \right)=0$$

so that all similar solutions put *m *= 2*n *in Equation 11, which becomes:

(12) $$\frac{1}{\text{pr}}\left(\frac{k_{nf}}{k_{f}}\right)\frac{\theta^{\prime \prime }}{k_{0}}+\frac{Ec}{{\left(1-\phi \right)}^{2.5}}f^{\prime \prime 2}+\left[\left(1-\phi \right)+\phi \frac{{\left(\rho c_{p}\right)}_{s}}{{\left(\rho c_{p}\right)}_{f}}\right]\left(f\theta^{\prime }-\frac{4n}{n+1}f^{\prime }\theta \right)=0,$$

and the transformed boundary conditions (Equation 4) become:

(13) $$\begin{array}{c} f\left(0\right)=0,\;f^{\prime }\left(0\right)=1,\;\theta \left(0\right)=1,\\ f^{\prime }\left(\infty \right)\to 0,\;\theta \left(\infty \right)\to 0, \end{array}$$

where Pr = *υ_f_/α_f _*is the Prandtl number, and *Ec *= *u_w_*^2^/[(*c_p_*)*_f_*(T_w _- T_∞_)] is the Eckert number. In the above equations, primes denote differentiation with respect to *η*.

It is worth mentioning that Equation 10 with the boundary conditions in Equation 13, with *n *= 0, is the classical Blasius flat-plate flow problem, and a detailed numerical study of that problem has been carried out by the author of this work. For the linearly stretching boundary problem (i.e., *n *= 1), the exact solution for *f *is *f*(*η*) = 1 - e^-*η*^; this exact solution is unique, while for the nonlinearly stretching boundary problem (i.e., n ≠ 1), there is no exact solution. The quantities of practical interest in this study are the skin friction coefficient *C_f _*and the local Nusselt number *Nu_x_*, which are defined as:

(14) $$C_{f}=\frac{2\mu_{nf}}{\rho_{f}{\left(u_{w}\left(x\right)\right)}^{2}}{\left(\frac{\partial u}{\partial y}\right)}_{y=0};\;Nu_{x}=\frac{-k_{nf}{\left(\frac{\partial T}{\partial y}\right|}_{y=0}x}{k_{f}\left(T_{w}-T_{\infty }\right)}.$$

Using Equation (9), the quantities (14) can be expressed as:

(15) $$\sqrt{\frac{C}{2\upsilon_{f}}}C_{f}=\frac{\sqrt{n+1}}{{\left(1-\phi \right)}^{2.5}}x^{-\frac{n+1}{2}}f^{\prime \prime }\left(0\right)$$

(16) $$\sqrt{\frac{2\upsilon_{f}}{C}}Nu_{x}=-\frac{k_{nf}\sqrt{n+1}}{k_{f}}x^{\frac{n+1}{2}}\theta^{\prime }\left(0\right).$$

## Results and discussion

**Figure 2:**
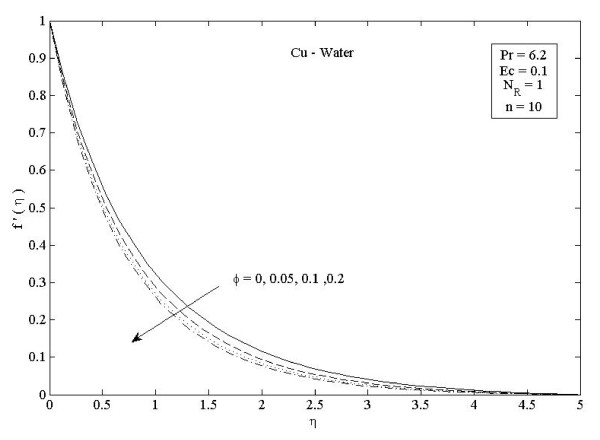
**Effects of nanoparticle volume fraction *ϕ *on velocity distribution *f*'(*η*) in the case of Cu-water**.

**Figure 3:**
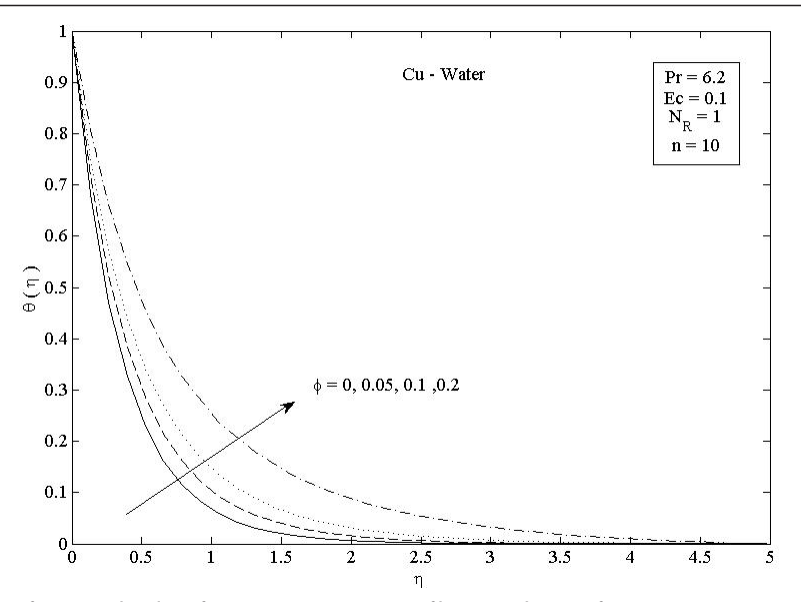
**Effects of nanoparticle volume fraction *ϕ *on temperature profiles *θ*(*η*) in the case of Cu-water**.

**Figure 4:**
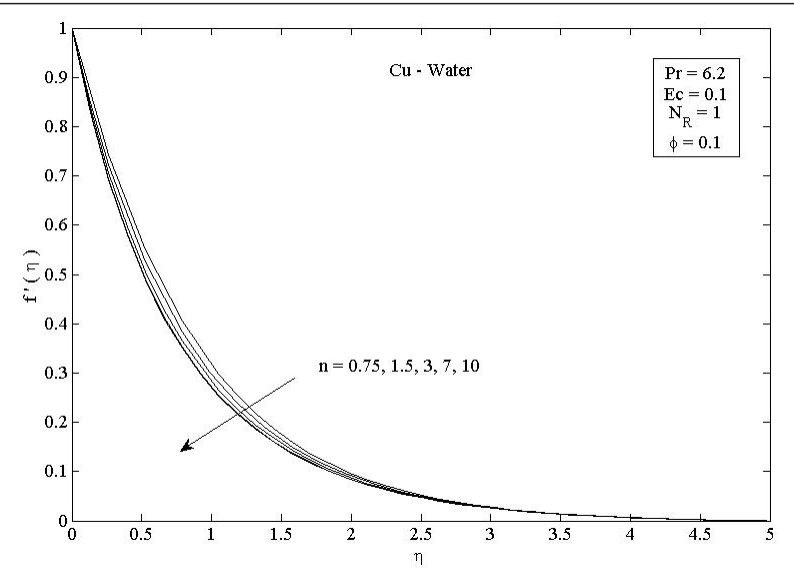
**Parameter *n *on velocity distribution *f*'(*η*) in the case of Cu-water**. Effects of nonlinearly stretching sheet parameter *n *on velocity distribution *f*'(*η*) in the case of Cu-water.

**Figure 5:**
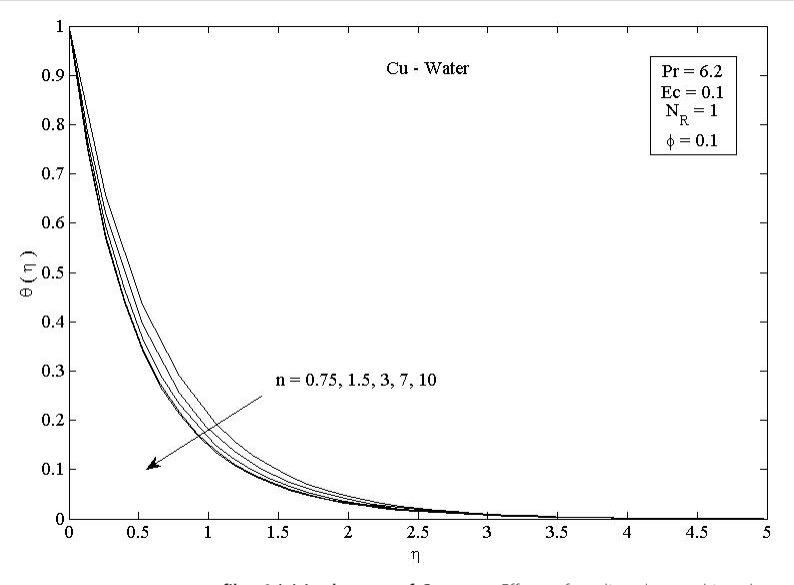
**Parameter *n *on temperature profiles *θ *(*η*) in the case of Cu-water**. Effects of nonlinearly stretching sheet parameter *n *on temperature profiles *θ*(*η*) in the case of Cu-water.

**Figure 6:**
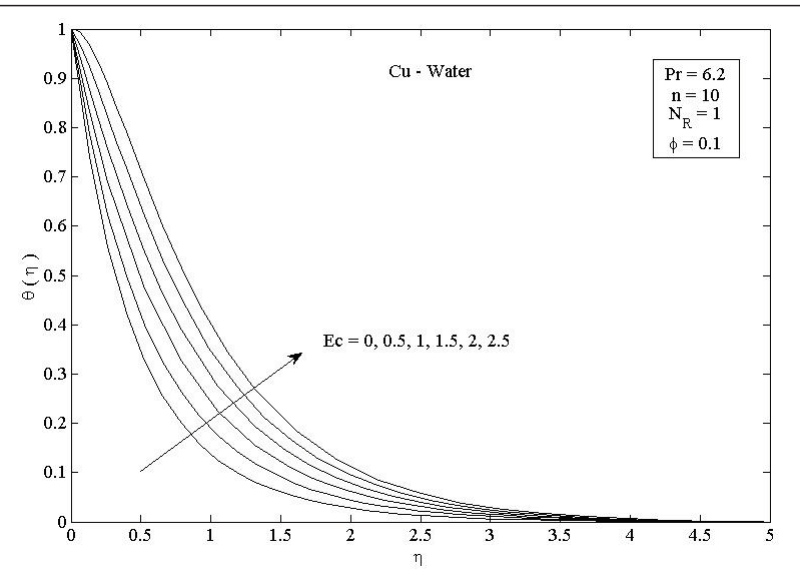
**Effects of viscous dissipation parameter *Ec *on temperature profiles *θ *(*η*) in the case of Cu-water**.

**Figure 7:**
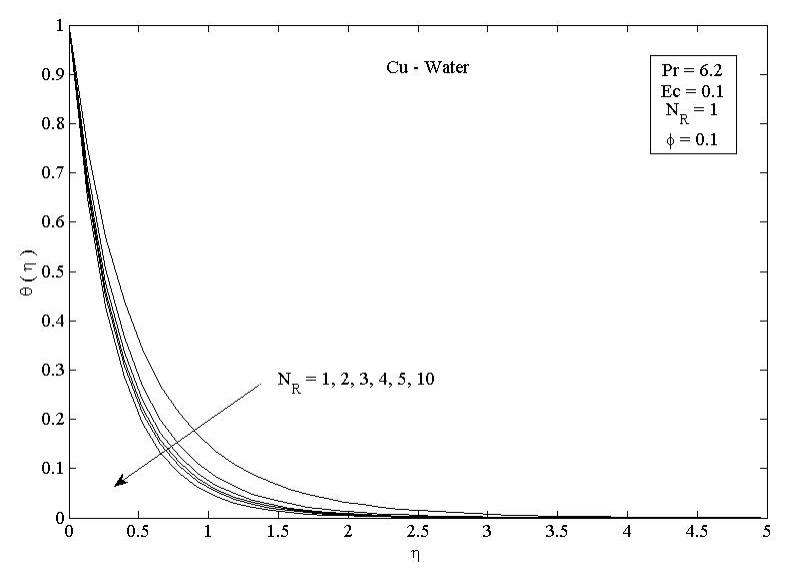
**Effects of thermal radiation parameter *N_R _*on temperature profiles *θ *(*η*) in the case of Cu-water**.

**Figure 8:**
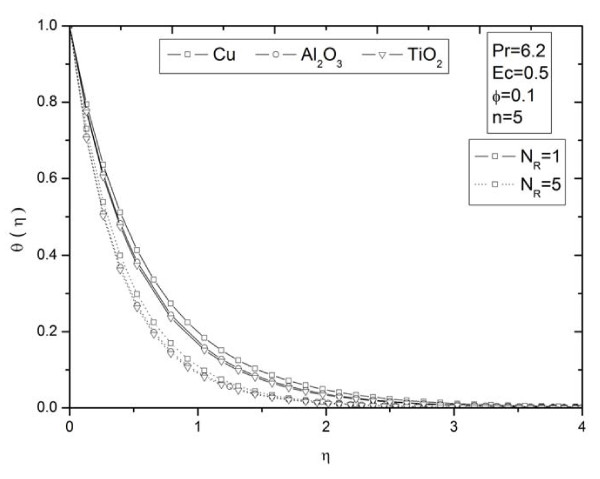
**Effects of thermal radiation parameter *N_R _*on temperature profiles *θ *(*η*) for different types of nanoparticles**.

**Figure 9:**
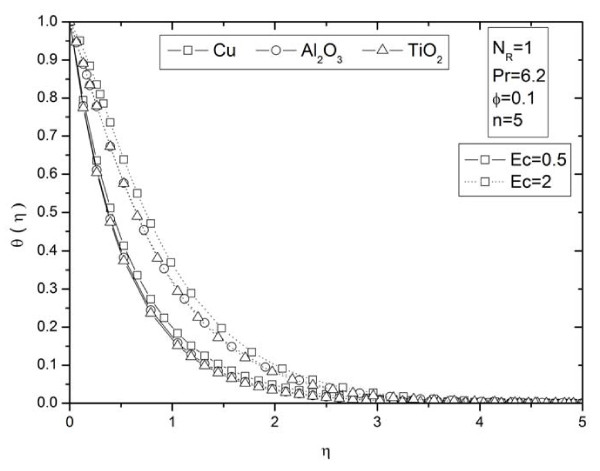
**Effects of viscous dissipation parameter *Ec *on temperature profiles *θ *(*η*)for different types of nanoparticles**.

**Figure 10:**
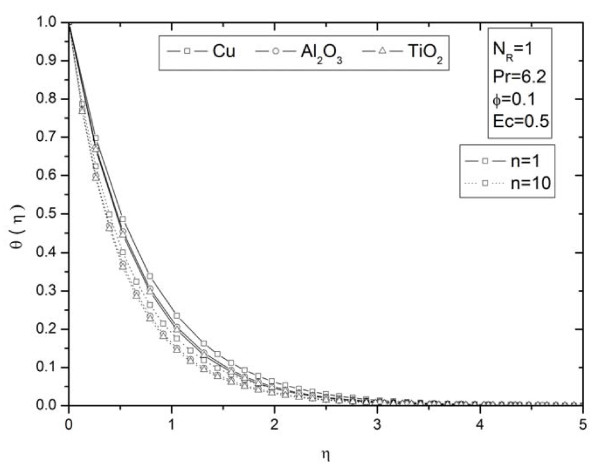
**Parameter *n *on temperature profiles *θ *(*η*)for different types of nanoparticles**. Effects of nonlinearly stretching sheet parameter *n *on temperature profiles *θ *(*η*) for different types of nanoparticles.

**Figure 11:**
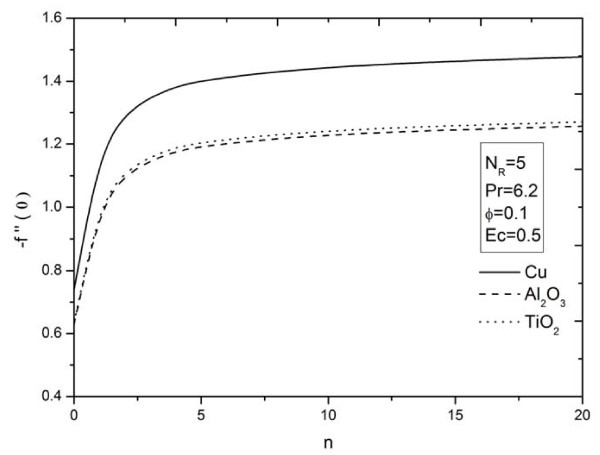
**Parameter *n *on skin friction coefficient for different types of nanoparticles**. Effects of nonlinearly stretching sheet parameter *n *on skin friction coefficient for different types of nanoparticles.

**Figure 12:**
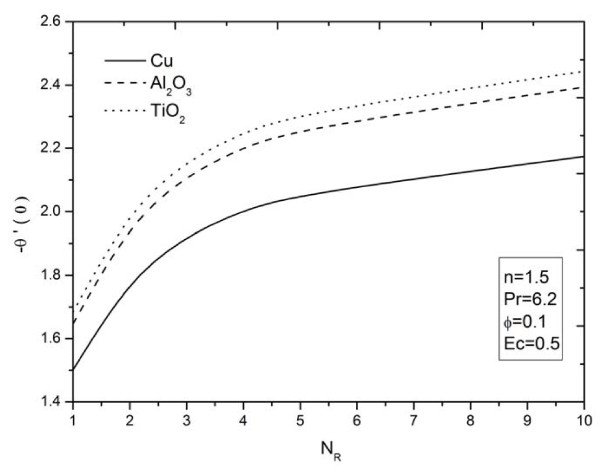
**Effects of thermal radiation parameter *N_R _*on heat transfer rate for different types of nanoparticles**.

**Figure 13:**
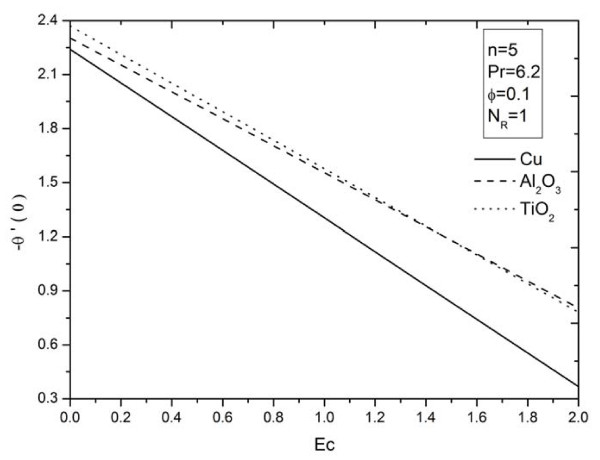
**Effects of viscous dissipation parameter *Ec *on heat transfer rate for different types of nanoparticles**.

**Figure 14:**
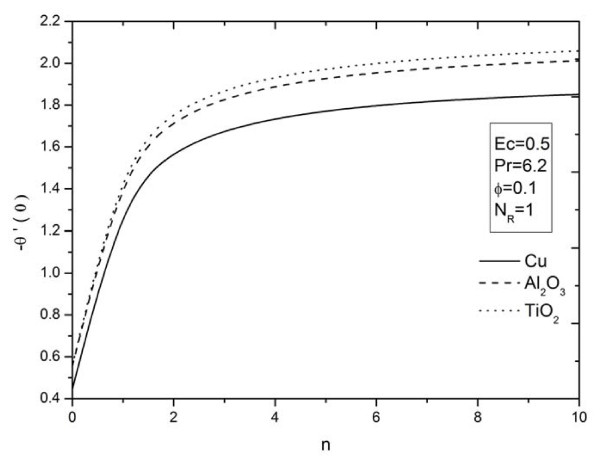
**Parameter *n *on heat transfer rate for different types of nanoparticles**. Effects of nonlinearly stretching sheet parameter *n *on heat transfer rate for different types of nanoparticles.

In order to get the physical insight into the flow problem, comprehensive numerical computations are conducted for various values of the parameters that describe the flow characteristics, and the results are illustrated graphically. The system of nonlinear ordinary differential Equations 10 and 12 with the boundary conditions (Equation 13) are integrated numerically by means of the efficient numerical shooting technique with a fourth-order Runge-Kutta scheme (MATLAB package). The step size *η *= 0.001 was used while obtaining the numerical solution with *η*_max _= 6. The physical quantities of interest here are the skin friction coefficient *C_f _*and the Nusselt number *Nu_x_*, which are obtained and given in Equations 15 and 16. The distributions of the velocity *f'(η)*, the temperature *θ*(*η*) from Equations 10 and 12, the skin friction at the surface, and the Nusselt number for different types of nanofluids are shown in Figures 2,3,4,5,6,7,8,9,10,11,12,13,14.

We consider three different types of nanoparticles, namely, copper (Cu), alumina (Al_2_O_3_), and titanium oxide (TiO_2_), with water as the base fluid. Table 1 shows the thermo-physical properties of water and the elements Cu, Al_2_O_3_, and TiO_2_. The Prandtl number of the base fluid (water) is kept constant at 6.2. It is worth mentioning that this study reduces the governing Equations 10-12 to those of a viscous or regular fluid when *ϕ *= 0. In order to verify the accuracy of the present method, we have compared our results with those of Cortell [12,13] for the rate of heat transfer - *θ'*(0) in the absence of the nanoparticles (*ϕ *= 0), without (*N_R _*→ ∞ (i.e., k_0 _= 1)) and with thermal radiation parameter. The comparisons in all the above cases are found to be in excellent agreement, as shown in Tables 2 and 3. Table 4 depicts the skin friction at the surface - *f*"(0) for various values of nonlinear stretching sheet *n*, with *ϕ *= 0.1, Pr = 6.2, *Ec *= 0.5, and *N_R _*= 5 for different types of nanoparticles when the base fluid is water. It can be seen from Table 4 that |*f*"(0)| increases with an increase in the nonlinear stretching parameter *n*, and the Cu nanoparticles are the highest skin friction, followed by TiO_2 _and Al_2_O_3_.

**Table 2:** Comparison of - *θ*' (0) with *ϕ *= 0 and *N_R _*→ ∞ (i.e., *k*_0 _= 1).

			**- *θ*'(0)**		
		
***Ec***	***n***	**Pr = 1**		**Pr = 5**	
		
		**Cortell **[12]	**Present study**	**Cortell **[12]	**Present study**
		
0.0	0.75	1.252672	1.253454	3.124975	3.123518
	1.5	1.439393	1.439378	3.567737	3.566532
	7	1.699298	1.698781	4.185373	4.184386
	10	1.728934	1.728383	4.255972	4.254939
	0.75	1.219985	1.220285	3.016983	3.013524
0.1	1.5	1.405078	1.404805	3.455721	3.453154
	7	1.662506	1.661742	4.065722	4.063757
	10	1.691822	1.691031	4.135296	4.133338

**Table 3:** Comparison of - *θ'*(0) for various values of thermal radiation parameter *N_R _*with *ϕ *= 0 (regular fluid).

***N***_***R***_	Pr	*Ec*	*n*	*-θ*'(0)	
				**Cortell **[13]	Present study
			1.5	-	0.832709
		0.05	3	-	0.923306
1	1		10	-	1.011487
		
			1.5	0.823356	0.824127
		0.1	3	0.913773	0.914364
			10	1.001573	1.002161
		
			1.5	-	0.755467
		0.5	3	-	0.842838
			10	-	0.927554
	
			1.5	1.295677	1.295790
		0.05	3	-	1.429987
			10	-	1.560471
		
			1.5	1.280575	1.280680
	2	0.1	3	-	1.414247
			10	-	1.544069
		
			1.5	1.159542	1.159609
		0.5	3	-	1.288335
			10	-	1.412856
	
		0.05		-	2.209436
	5	0.1	1.5	2.178778	2.178846
		0.5		-	1.934126

		0.05		-	1.584762
2	2	0.1	1.5	1.564987	1.565049
		0.5		-	1.407369

		0.05		-	1.925487
5	2	0.1	1.5	1.833888	1.834037
		0.5		-	1.639374

**Table 4:** Values related to the skin friction for different values of n.

*n*		-*f*"(0)	
	
	Cu	**Al**_**2**_**O**_**3**_	**TiO**_**2**_
0	0.737218	0.626792	0.633534
1	1.174748	0.998779	1.009523
2	1.293408	1.099665	1.111494
3	1.349309	1.147192	1.159532
4	1.381883	1.174886	1.187525
5	1.403223	1.193030	1.205863
10	1.450669	1.233367	1.246635
20	1.477159	1.255889	1.269399
50	1.494071	1.270267	1.283932
100	1.499890	1.275215	1.288933

With *ϕ *= 0.1, Pr = 6.2, *Ec *= 0.5, and *N_R _*= 35.

Figures 2 and 3 illustrate the effect of nanoparticle volume fraction *ϕ *on the nanofluid velocity and temperature profile, respectively, in the case of Cu nanoparticles and water base fluid (Pr = 6.2) when *ϕ *= 0, 0.05, 0.1, and 0.2, with *Ec *= 0.1, *n *= 10, and *NR *= 1. It is clear that, as the nanoparticles volume fraction increases, the nanofluid velocity decreases, and the temperature increases. These figures illustrate this agreement with the physical behavior. When the volume of nanoparticles increases, the thermal conductivity increases, and then the thermal boundary layer thickness increases. Figures 4 and 5 depict the effect of nonlinearly stretching sheet parameter *n *on velocity distribution *f*'(*η*) and temperature profile *θ*(*η*), respectively. Figure 4 illustrates that an increase of nonlinear stretching sheet parameter *n *tends to decrease the nanofluid velocity in the case of Cu-water when *n *= 0.75, 1.5, 3, 7, and 10, with *Ec *= 0.1, *N_R _*= 1, and *ϕ *= 0.1. Furthermore, Figure 5 shows that increasing the nonlinear stretching sheet parameter *n *tends to decrease the temperature distribution the same values, thus leading to higher heat transfer rate between the nanofluid and the surface. The effect of the viscous dissipation parameter *Ec *on the temperature profile in the case of Cu-water when the Eckert number *Ec *= 0, 0.5, 1, 1.5, 2, and 2.5 with *n *= 10, *N_R _*= 1, and *ϕ *= 0.1 is shown in Figure 6. It is clear that the temperature distribution increases with an increase in the viscous dissipation parameter *Ec*. Figure 7 shows the influence of thermal radiation parameter *N_R _*on the temperature profile in the case of Cu-water. It is clear that the temperature decreases with an increase in the thermal radiation parameter *N_R_*; this leads to an increase in the heat transfer rate. Moreover, Figure 8 shows this effect of the thermal radiation parameter on the temperature distribution but for the different types of nanoparticles with water as the base fluid. It can be seen from Figure 8 that *θ*(*η*) decreases with an increase in the thermal radiation parameter as shown in Figure 7, and the Cu nanoparticles have the highest value of temperature distribution than the nanoparticles Al_2_O_3 _and TiO_2_. The influence of *Ec *and *n *on the temperature profiles for all types of nanoparticles is shown in Figures 9 and 10, respectively. It is found that the temperature decreases with *n *and increases with *Ec *as shown in Figures 5 and 6, respectively, and the TiO_2 _nanoparticles proved to have the highest cooling performance for this problem.

The influence of nonlinear stretching sheet *n *on the skin friction at the surface -*f'' *(0) with *N_R _*= 5, Pr = 6.2, *ϕ *= 0.1, and *Ec *= 0.5 is shown in Figure 11. It can be noticed that, from Table 4 and Figure 11, the numerical values of |*f'' *(0)| for different kinds of nanofluids increase with an increase in the nonlinear stretching parameter *n*. This implies an increment of the skin friction at the surface where Cu nanoparticles have the highest skin friction than the other nanoparticles. Figures 12,13,14 display the behavior of the heat transfer rates under the effects of *N_R_, Ec*, and *n*, respectively, using different nanofluids for Pr = 6.2 and *ϕ *= 0.1. These figures show that, when using different kinds of nanofluids, the heat transfer rates change, which means that the nanofluids will be important in the cooling and heating processes. It can be noticed from the results above that, as expected, the heat transfer rate increases with an increase in the thermal radiation parameter *N_R _*and nonlinear stretching sheet parameter *n*, and decreases rapidly with an increase in the viscous dissipation parameter *Ec*.

## Implications of the hypothesis

The problem of boundary-layer flow and heat transfer in a viscous nanofluid over a nonlinearly stretched non-isothermal moving flat surface in the presence or absence of thermal radiation using the Rosseland approximation for the radiative heat flux was analyzed. The governing partial differential equations were converted to ordinary differential equations by using a suitable similarity transformation and were then solved numerically via shooting method by employing throughout our calculations the fourth-order Runge-Kutta scheme (MATLAB package). The effects of the solid volume fraction *ϕ*, thermal radiation parameter *N_R_*, nonlinear stretching sheet parameter *n*, and the viscous dissipation parameter *Ec *on the flow and heat transfer characteristics are determined for three kinds of nanofluids: copper, alumina, and titanium oxide.

1. The increase of the solid volume fraction *ϕ *and the nonlinear stretching sheet parameter *n *leads to the decrease of dimensionless surface velocity; this yields an increase in the skin friction at the surface.

2. An increment in the solid volume fraction *ϕ *and the Eckert number *Ec *yields an increment in the nanofluid's temperature; this leads to a rapid reduction in the heat transfer rates.

3. An increase in the thermal radiation parameter *N_R _*and the nonlinear stretching sheet parameter *n *yields a decrease in the nanofluid's temperature, which leads to an increase in the heat transfer rates.

4. The TiO_2 _nanoparticles proved to have the highest cooling performance for this problem than the other two types of nanoparticles (cu and Al_2_O_3 _nanoparticles).

## Abbreviations

### Nomenclature

*b*: constant; *C*: physical parameter related with stretched surface; *C_f_*: skin friction coefficient; *c_p_*: specific heat; *Ec*: Eckert number; *f*: dimensionless stream function; *k*: thermal conductivity; k*: mean absorption coefficient; *m*: surface temperature parameter; *N_R_*: radiation parameter; *Nu_x_*: Nusselt number; Pr: Prandtl number; *q_r_*: radiative heat flux; *n*: nonlinear stretching parameter; *T*: temperature; *u *and *v*: velocity components along *x*- and *y*-directions: respectively; *x *and *y*: Cartesian coordinates along the plate and normal to it: respectively.

### Greek symbols

*α*: thermal diffusivity; *η*: similarity variable; *θ*: dimensionless temperature; *μ*: effective viscosity; *υ*: kinematic viscosity; *ρ*: density; σ*: Stefan-Boltzmann constant; (*ρC_p_*)*_nf,_*heat capacitance of the nanofluid; (*ρC_p_*)*f*: heat capacity of the fluid; (*ρC_p_*)*_s_*: effective heat capacity of the nanoparticle material; *ϕ*: nanoparticle volume fraction.

### Subscripts

*f*: fluid fraction; *nf*: nanofluid fraction; *s*: solid fraction; *w*: condition at the wall; **∞**: stream function condition at infinity.

## Competing interests

The authors declare that they have no competing interests.

## Authors' contributions

MRE did the major part of the article; however, the funding, computational suggestions, and proof reading were done by FSI, FMH and SMAG. All authors read and approved the final manuscript.

## Authors' information

FMH and FSI are professors of applied mathematics. SMAG is a mathematics lecturer, and MR is a PhD student.

## Endnotes

This is just a theoretical study; every experimentalist can check it experimentally with our consent.
